# Synopsis of Yellow Fever, with Its Pathology and Treatment, as It Appeared at the Marine Hospital, Quarantine, N. Y., during 1854 and 1855

**Published:** 1856-04

**Authors:** Theod. Walser

**Affiliations:** Assistant Surgeon


### THE NORTH-WESTERN

### MEDICAL AND SURGICAL JOURNAL.

(NEW SERIES.)

VOL. V. APRIL, 1856. NO. 4.

### ORIGINAL COMMUNICATIONS.

ARTICLE I.—*Synopsis of Yellow Fever, with its Pathology and Treatment, as it appeared at the Marine Hospital, Quarantine, N.Y. during* 1854 *and* 1855.—By THEOD. WALSER, M.D., Assistant Surgeon.

The first cases of yellow fever were received by the Empire City, from Havana, when four patients were admitted July 4, of whom two died and two were discharged. All of these were Americans, and residents in Cuba during the prevalence of the epidemic there, and to escape which they embarked for home. No more were received until the Augusta, from Savanah, anchored on August 29. She had lost one man, a passenger, during her voyage, and eight more sent to the Marine Hospital with yellow fever, during the time of her Quarantine. Only a few days afterwards, the Alabama, the Knoxville, the Florida, and subsequently the James Adyer, the Pocahontas, the Southener, the Columbia, all from Savannah or Cuba, left in altogether fifty cases in our care, of whom twenty-four died and twenty-six were discharged. During the whole summer, no other cases occurred in this Hospital or on board of any vessel but those named above who came from an infected part. We except, however, a New York longshoreman, who was engaged in unloading the cargo of an infected vessel, and who, while on board, was attacked with the disease and died of yellow fever, although he had never left New York. In 1855, ten cases came under our observation and treatment; five of these were received from vessels from New Orleans, two from small coasters between James River and New York, two more of railroad from Norfolk, and one from the United States ship Falmouth, from Key West. All were sailors with the exception of two, who came by railroad. The first five were ashore in New Orleans or Cuba, and attacked in from one to five days after leaving port; the two from the coasters never came nearer Norfolk than six miles, the wind blowing from that direction; of the two cases received by railroad, one, Mountani, was attacked immediately after arriving in New York, and the other, three days after leaving Norfolk. Among those whom we received here, we regret that very few came under our treatment at the commencement of the disease—almost all were in an advanced stage already, and many were admitted in a hopeless condition.

From what we could learn of the patient, and from what we observed, we had premonitory symptoms only in a few cases, and then of such character that they could scarcely be defined. In almost all the cases the disease was suddenly ushered into existence with pain in the head, pain in the back of the neck, and small of the back, languor, &c. Sometimes with a short chill, but more frequently not even with this. The attack was generally so sudden and so violent, that any further labor and exertion became impossible, and scarcely was the patient able to continue his usual occupation even a day after its onset. The character of the disease manifested itself further, in an excited, frequent, full and strong pulse, injection of the conjunctiva, the weary eye suffused with tears, the prominent, lustrous cornea, the coated tongue, first thickened and white, afterwards whitish, with clean red tip and edges, the flushed face, hot skin, and general debility.

After the lapse of the first twenty-four hours, a slight remission ensues, headache partially disappears, the pain becomes somewhat milder, the injected eye loses its lustre somewhat, but to the careful observer, the brilliant conjunctiva is slightly tinged with sulphur-yellow, the red tip and edges of the now more thickly coated tongue become more distinctly visible, the pulse becomes feebler and often slower, a slight heaviness in the epigastric region, pain on pressure, a morbid excitability or restlessness, and at the same time a general debility and weakness follow the first attack, which scarcely admit this so called stage of remission to be considered an amelioration of the symptoms.

So uniform as the initiatory symptoms of yellow fever may be, so widely will they differ after a lapse of twenty-four or thirty-six hours—for in the more malignant forms, black vomit may then set in, and the patient in a few hours more is a yellow corpse; in others, not so rapid in their course, the same order of symptoms continues with scarcely any alteration, for two, three or six successive days, when suddenly the patient is taken with a chill, and in an hour or two he is a corpse. Among these latter cases, I remember particularly a Norwegian sailor, sick for four days—pulse 92, full—his eye but slightly tinged, and only a remaining dulness and stupidity, warning the physician of the danger—he was suddenly taken with a violent chill—shivering as if with common ague—an hour afterwards he was a yellow corpse—the autopsy revealing all and only the characteristics of yellow fever. In other cases, again, the patient becomes more dull and stupid—indifferent to himself and all around him—his pulse remains from 100 to 120—full, but irregular. The watery eye becomes by degrees more and more yellow, until the conjunctiva presents a deep orange color. The surface of the body itself gradually participates in this discoloration—the secretion of urine is scanty, yellow, and oil-like—the gums begin to bleed—the tongue resembles raw beef, coated with purple blood—thick, black sordes cover the teeth, the upper eyelids become gradually blue, (ecchymosed)—small purple spots of the size of a hemp seed (ecchymosis) appear, chiefly on the face, breast, and back. Black vomit or egurgetation of a copious, brownish mass (vomito) takes place—stools even participate of the character of black vomit—copious, bright, black, gelatinous evacuations follow; his eyes, before watery, now exude a bloody serum—his nose, his ears begin to bleed—until, indifferent to life or death, the patient sinks to his grave.

But not all the cases which we have had the misfortune to lose, died in this manner; some retained their power of mind almost entire. After a few days, the initiatory symptoms and pains leave the patient, and only restlessness, want of sleep, and a general uneasiness remains—his pulse remains quick—his eye deepens its yellow hue. The patient is able to converse on all and every topic rationally—his animal propensities may even be increased, but still the experienced physician sees in his eye and face, death lurking, and soon, too soon, finds his fears verified in the black vomit, the never-failing precursor of approaching death. But even this does not concern the patient, he ejects it as tobacco juice, with equally as much ease and force, until suddenly and unexpectedly he dies. Others, with the same tranquility of mind, and the same symptoms, show approaching dissolution in the bleeding gums, mouth, ears, and nose. The prognosis of an uninterrupted convalesence, could only be made in five or six days after the first attack of the disease, although even at an earlier period we may reasonably entertain hopes of a favorable issue. In these cases the pain in the back and head gradually disappears, the mind becomes clearer and more rational—the pulse becomes slower, but feeble and regular, falling, in most cases, even far below the normal standard, (a symptom which I never observed in any case that ended fatally). The tongue changes to a more uniform color, the red tip and edges disappearing—the secretion of urine becomes more free and copious, mostly leaving a whitish sediment on the bottom of the vessel—his excrements are brown, feculent, and semi-fluid. The physiognomy loses its stupid and drunken appearance—the yellowness of the skin and eyes, never very intense, gradually disappears—the patient desires to eat, relishes his food, and after ten or twelve days may be considered out of danger. In few cases only the convalescence is prolonged to a much longer period.

POST-MORTEM APPEARANCES.

In all the cases the body was of an intense yellow, more so than during the last moments of the patient's life; the lower depending parts of a bluish-purple, sprinkled with round, bluish black spots (sugillations) of the size of a pin's head, less marked, and frequently entirely wanting on the upper surface —the scrotum, the ears almost black—frequently a dark blood flows from the mouth and nose—the body retains its heat for a longer time than usual—when cold, it becomes rigid—upon incision, the fatty covering appears of an intense yellow color—the muscles are dark—the opened vessels bleeding—the flowing blood almost black, (brownish-black).

*Head.*—The incised scalp bleeds freely—after removing the cranium, the dura mater found thickly coated with blood, drops of which appear dark blue. The suljacent arachnoidea is so intensely congested, that the brain appears covered with blood, and in some places even amounting to extravasation of one or two lines thickness. By removal of the brain, from ℥ij. to ℥iv. of bloody yellowish serum escape from the base of the brain, which thereby being less congested, presents the peculiar appearance of a floating cobweb, if a stream of water be poured upon the serous investment of its basial portion. In one instance only, we found this congestion less intense, and in that case, the patient was treated with cups and the continued application of ice to the head. The substance of the brain is universally normal—the incision followed with innumerable points of blood—the ventricles empty—the plexus choroides congested. This state of things was so uniform, that I know of no exception.

*Thorax.*—In the opening of the thorax, care had to be taken not to open the larger vessels, as these almost always bled copiously and profusely. The lungs were in sixteen out of twenty cases normal, the lower part usually slightly congested, and in a few cases the surface of the lungs was covered with dark spots, of the size of a pea to a hazel nut; these spots corresponding to round, well-defined spleen-like spots in the parenchyma of the lungs, and only observed towards the periphery, and never towards the centre of the lungs. The costal pleura always congested and reddish—in the pleural cavity itself, in six cases, from a half to one pint of fluid blood was found, the blood being more serous in its character than is natural, evidently the result of transudation through the costal pleura—the inner lining of the larger vessels, brick-red and deeply injected. The heart, in the most malignant cases, flabby and soft in its texture—the auricles and the ventricles filled with partially coagulated blood, but in no case could we find the fibrinous clot. The endocardium was in six cases covered with vibices, or there was an extravasation of blood into the cardiac structure,, immediately beneath the endocardium, since, on removal, this membrane appeared normal in character. In the pericardium was found, generally, from ℥i. to ℥iv. of yellow serum—the membrane itself, normal and healthy.

*Abdomen—Liver.*—The liver was in all cases a bright, uniform yellow, and on section, somewhat less yellow internally—of normal size and consistence, and slightly fatty; in only a few cases we found it softened, and then I attributed it to incipient decomposition. The lower surface of the liver was uniformly of a purplish-blue, this color extending only one or two lines in depth. The gall-bladder filled with a semi-fluid, greenish-brown bile—the mucous membrane of the gall-bladder slightly congested. *Spleen* was in all cases normal, rather of a brownish-red cast, otherwise, exhibiting nothing unusual. The kidneys were normal, the cortical substance generally yellowish and pale—the pyramids unusually distinct. Bladder generally empty, or containing very little yellowish acid urine.

*Stomach and Intestines.*—In almost every case we found the stomach to contain black vomit or blood, no matter whether the patient vomited it before his death or not. In two cases we found it full of blood, these having taken large doses of soda bicarb. before they died. The mucous membrane of the stomach appeared, in some cases, of a greyish color, in more cases it was of a brownish hue, and in a few cases it was found somewhat softened and thickened; the softening, however, not of sufficient degree and character to warrant the diagnosis of inflammation. About the pyloric extremity most frequently, but in a few instances towards the cardiac orifice, a large number of minute red spots could be seen, apparently radiating from a centre, and having an arborescent appearance. I found no other constant lesions. In one case only was there ulceration and entire removal of the mucous membrane, to the extent of a three cent piece, in three or four different points, and in this case no black vomit was found in the stomach, the patient having died, some fifteen days after the first attack of the disease, of a typhoid (continued) fever. In a few cases, ecchymosis was found in the stomach.

The duodenum and jejunum were generally of a uniform appearance with the stomach—much injected—in some few instances so much so, that they appeared of a blood-red color, in which the individual vessels scarce could be distinguished; their contents were the same as the contents of the stomach, only mixed with more mucus and bile, and they seemed to have changed their character from black vomit to a semi-fluid, gelatinous and homogeneous mass, of a chocolate color. Brunner's glands and the solitary glands were universally enlarged and elevated. In the ileum was found the same blackish mass, intermixed with which small portions of fæcal matter might occasionally be observed, which had lost neither its peculiar color or appearance.

In the colon and rectum, the mucous membrane appeared merely congested, and their contents the same as in the small intestines, only with a larger admixture of fæcal matter.

I have thus given a summary of all our cases, none deviating from the general type, although the one or the other may have differed in an individual particular, or in having one or the other lesion more developed.

*Treatment.*—Immediately after the first onset of the disease, if on board or ashore, physicians and patients were equally anxious to meet the approaching enemy with a brisk cathartic of calomel—only in some six or eight cases, a large dose of ol. ricini was administered in its place.

Although no one can doubt the expediency of giving a cathartic at the onset of the disease, it is certainly no way clear in my mind which of the two deserves preference. If we consider the peculiar yellow tinge, that the conjunctiva and afterwards the body assume—the fact which I repeatedly tested, that in an advanced stage of yellow fever the peculiar coloring matter of the bile may be detected in the serum obtained from the pericardium after death, or from the serum of a blister during the stadium depressionis—if we consider also the engorged state of the liver, which we invariably find in our autopsies, we may certainly find a reason for prefering calomel to promote the secretion of bile, and to relieve the liver of its congestion. But if we consider, on the other hand, the peculiar dissolution of the blood, the tested fact, that in yellow fever the blood loses its fibrine and its tendency to coagulate, in consequence of which the capillaries are unable to retain the blood in its proper channels, and transudation necessarily ensues in all vascular, and particularly in all serous membranes, then we hesitate to employ an agent whose acknowledged virtue is to deprive the blood of its tendency to fibrinization and organization; besides this, I am by no means convinced that this structural change of the liver is primary. On the contrary, from the fact that the initiatory symptoms necessarily must arise from a peculiar morbid state of the nervous systems and their centers, viz: the brain and solar plexus, with consequent congestion, manifest in the injected cornea, wakefulness and uneasiness, fulness in the epigastrium and pain in the back; and again, from the fact that the peculiar coloring matter cannot be detected in the stadium invasionis. I am more and more convinced that this peculiar change in the liver is dependent upon this morbid state of the nervous system, and consequent dissolution of the blood. The reason why we find at a later period, the coloring matter of the bile in the serum of the blood, may be attributed to the universal suppression of all normal secretion, and the yellow discoloration of the skin and conjunctiva may partly arise from the presence of the coloring matter of the retained bile, and partly from extravasation and undue proportion between arterial and venous blood. Perhaps this may be more clearly expressed by saying that during the exhalation from the capillaries, hæmatine, changed from its normal condition, is exuded into the areolar tissue, and there produces the yellow discoloration. That this exhalation is changed in its character, we have ample evidence in the deep yellow serum, somewhat blood-like in character, found in all the serous membranes arising from the extravasation of the disorganized blood; and that a blood-like extravasation, if imperfectly absorbed, produces this yellow color, we see in the yellow patches of purpura hæmorrhagica and in bruises coming every day under our observation.

As I observed, the first remedies employed were calomel or ol. ricini; each one operating as cathartic, and producing copious brownish black stools.

In four cases, after the cathartic, only soda bicarb. was used. In one only can I report a happy result. In others it had the effect of changing the black vomit to bright red blood. In the autopsies of these cases, much blood was found in the stomach, and also, to some extent, in the intestines.

In all other cases, large doses of quiniue were administered immediately after the stadium invasionis and the operation of the cathartic. This treatment was continued for several successive days, and careful and diligent observation leads me to believe *that whatever success we had in our treatment, we owe it to this remedy alone, at this early stage.* The use of quinine in yellow fever has as many and powerful advocates as adversaries, and although the view of a miasmatic cause impressing the nervous system, and producing the above-named pathological results, absolutely demands its employment, I am, nevertheless, not entirely satisfied that these views are correct, or that this peculiar nervous depression is dependent upon that ill-defined cause which we express by the vague term of *miasm.* I had often opportunities to compare the close resemblance of the initiatory symptoms of pernicious, remittent, and yellow fever, and more particularly the afore-mentioned fact, that yellow fever frequently terminates in an actual paroxysm, immediately followed by death; all of which might induce me to classify yellow, remittent, and pernicious fever under the general head of miasmatic fevers, and therefore employ the same "specific" for one and all. But if I consider the vast difference of the post-mortem appearances, no reasoning power, or even experience, could induce me to classify these diseases under one and the same head.

In remittent fever, the corpse is of a greenish-yellow, but in yellow fever it is of a deep yellow, with innumerable sugillations and ecchymoses, never found in the former. In intermittent fever, the blood is brick-red and watery; in pernicious fever, dark, with firm, fibrinous clots in the heart and larger vessels; in yellow fever, it is brownish-red, forming very imperfect coagula in the heart, and never a fibrinous clot; rarely can we find a vestige of a jelly-like substance. The spleen in intermittent, enlarged and solid; in remittent fever, enlarged and so soft that the fingers pass through it in the attempt to remove it; in yellow fever, it is small and normal. The liver of remittent fever, is bronze-colored, and enlarged and soft; in yellow fever, intense yellow, hard and small. I mention here only the principal pathological lesions of both diseases, but to my mind, sufficient to show the vast difference between yellow and remittent fever; the remedy for the one must not necessarily be the remedy for the other, or more probably, cannot be the remedy for the other. Yellow fever bears far more resemblance in its ultimate results to purpura hæmorrhagica, although the cause and the first symptoms differ widely from each other; this resemblance may also account for the use of tinct. ferri sesquichloridi, once so much in vogue during the epidemic in Savannah, in 1854.

Notwithstanding all this, I shall employ quinine in every case coming under my observation and treatment, until I find another agent better adapted to counteract this peculiar nervous impression, and to prevent the dissolution of the blood, with the consequent organic changes.

Our attention being further called to the constant lesion of the congestion of the brain; we applied leeches to the neck and head; and more particularly the ice-cap, which was universally followed by relief. Leeches to the epigastrium, so highly recommended by some writers, were also employed in a number of cases, and were generally followed by relief of the fulness of the epigastrium, and what was more remarkable, always relieved the pain in the back; care had to be taken with their application, as it was almost impossible to stop the bleeding.

Of unquestionable benefit I considered, in practice, never to allow the patient anything to eat during the first week of his illness; in two cases, where this strict rule was not observed, the patient's life was sacrificed in consequence.

I have endeavored to give a brief synopsis of our cases, with their pathology and therapeia; I am fully convinced of its imperfections, and more particularly as respects chemical analysis of the different secretions, and especially the blood, and also careful microscopical examinations of all the pathological products, so necessary to arrive at anything like a truthful result in our observations and studies; for only a thorough knowledge of the pathology of a disease can lead to a sound and rational therapeia.

